# The Effect of Whole-Body Cryotherapy at Different Temperatures on Proinflammatory Cytokines, Oxidative Stress Parameters, and Disease Activity in Patients with Ankylosing Spondylitis

**DOI:** 10.1155/2018/2157496

**Published:** 2018-10-03

**Authors:** Anna Straburzyńska-Lupa, Magdalena Paulina Kasprzak, Mateusz Wojciech Romanowski, Anna Kwaśniewska, Wojciech Romanowski, Maria Iskra, Radosław Rutkowski

**Affiliations:** ^1^Chair of Physical Therapy and Sports Recovery, Poznan University of Physical Education, Droga Dębińska 10C, 61-555 Poznań, Poland; ^2^Department of General Chemistry, Chair of Chemistry and Clinical Biochemistry, Poznan University of Medical Sciences, Rokietnicka 9, 60-806 Poznań, Poland; ^3^Department of Rheumatology and Rehabilitation, Poznan University of Medical Sciences, 28 Czerwca 1956 135/147, 61-545 Poznań, Poland; ^4^Rheumatological Centre in Śrem, Mickiewicza 95, 63-100 Śrem, Poland

## Abstract

**Purpose:**

Patients with ankylosing spondylitis (AS) have increased production of proinflammatory cytokines, increased oxidants, and decreased antioxidant capacity. The aim of this study was to determine the effect of whole-body cryotherapy (WBC) at −110°C and −60°C, on disease activity, selected proinflammatory cytokines, and oxidative stress in patients with AS.

**Methods:**

Sixty-five patients with AS were recruited to one of three study procedures: WBC at −110°C, −60°C, or exercise therapy (non-WBC). The Bath Ankylosing Spondylitis Disease Activity Index (BASDAI), Ankylosing Spondylitis Disease Activity Score (ASDAS-CRP), concentration of C-reactive protein (CRP), and the concentrations of interleukin 8 and 17 (IL-8, IL-17) were measured at the beginning of the study and at the end of the intervention. The concentration of thiobarbituric acid reactive substances (TBARS), as a lipid peroxidation result, and total antioxidant status, an antioxidant organism potential, were measured.

**Results:**

All the studied groups showed significantly decreased posttherapy disease activity expressed as a function of the BASDAI, ASDAS-CRP, and the IL-8 concentration. We found that the TBARS concentration after therapy was significantly increased in the WBC at −110°C group. A comparison of the therapeutic effects between the treatment groups showed a significantly lower BASDAI after therapy in the WBC at −110°C group compared to the non-WBC group.

**Conclusion:**

WBC at −110°C had a positive effect on lowering AS clinical activity as measured by the BASDAI.

## 1. Introduction

Ankylosing spondylitis (AS) is an autoimmune-related, chronic inflammatory rheumatic disease of unknown etiology. This condition typically occurs in men and mainly affects the axial skeleton and extra-articular structures. It may also involve the peripheral joints and specific organs like the eyes or the bowel [1, 2]. It has been demonstrated that there is increased bone formation at sites of inflammation in ankyloses [3]. Clinically AS is characterized by pain, reduced spine mobility, and functional impairments [1, 2]. The pathophysiology of AS is not fully recognized. It is suggested that genetic, immunological, and inflammatory factors are the most important in the onset of the disease [1–3].

Many proinflammatory cytokines and chemokines may be involved in the pathogenesis of autoimmune rheumatic diseases [4–6]. Wang et al. [7] showed elevated levels of IL-23 and IL-17 in the serum of patients with active AS. These findings drew attention to the potential central role of the IL-23/IL-17 axis in AS [8]. It has been shown that IL-17 enhances T-cell activation and stimulates various cell types (fibroblasts, endothelial and epithelial cells, and macrophages) to produce proinflammatory mediators (IL-1, IL-6, TNF-*α*, and chemokines) [9]. IL-17 has been implicated in chronic autoimmune diseases such as rheumatoid arthritis, sclerosis multiplex, psoriasis, and AS [10, 11]. Mei et al. [4] showed that serum IL-17 levels were significantly elevated in patients with AS compared to normal controls. However, its role in the pathogenesis of AS remains unclear. Aberrant production of the chemokine IL-8 can result in chronic inflammatory conditions, such as rheumatoid arthritis, psoriasis, and the inflammatory bowel diseases [12]. Limón-Camacho et al. showed that serum levels of IL-8 were significantly higher in patients with AS compared to controls [13]. Azevedo et al. suggested that IL-8 would be a good marker for disease activity in AS patients who did not receive anti-TNF agents [14].

The increased production of proinflammatory cytokines generally accompanies increased levels of oxidative stress markers [5]. Recent studies have reported an increase in oxidative stress in AS [15, 16]. AS increases oxidant processes and decreases antioxidant capacity of the system; however, the association of AS with oxidative stress markers and cytokines remains uncertain [5, 16, 17].

Physical therapy plays an important role in the long-term comprehensive treatment of patients with AS [1, 2]. Cryotherapy is often used as an additional form of therapy for the inflammatory rheumatic diseases [18, 19]. Cold relieves pain, and therefore, it enables the patient to intensify their exercise [20]. It is postulated that cryotherapy causes changes in the level of cytokines, which seem to affect the immune mechanisms in the body [21]. Guillot et al., in their expert review, concluded that cryotherapy might decrease the level of proinflammatory cytokines and probably could be used in RA and other joint inflammatory diseases as an adjunct therapy to disease-modifying antirheumatic drugs (DMARDs), targeted biologic treatments, corticosteroids, and nonsteroidal anti-inflammatory drugs (NSAIDs). This could lead to a reduction in the pharmacological treatment and minimize numerous well-known side effects associated with it [18]. A few research carried out in recent years confirmed the positive influence of cryogenic temperatures on patients with AS on inflammatory markers [22] and cytokines [23]. At the same time, intensive research is being conducted on the effect of whole-body cryotherapy on oxidative stress in healthy people [21, 24, 25] and AS patients [23, 26]. However, it is noted that the establishment of standardized and optimized treatment protocols requires further research [18].

Thus, the objective of this study was to evaluate the effects of a cycle of exposure to WBC at −110°C and WBC at −60°C on disease activity, level of proinflammatory cytokines, and oxidative stress in a group of patients with AS.

## 2. Materials and Methods

### 2.1. Participants

The study group comprised 65 patients with a diagnosis of AS according to the modified New York criteria who were hospitalized in the rheumatology ward [1, 2].

The inclusion criteria were BASDAI ≥ 4, on a stable medication regimen, nonsteroidal anti-inflammatory drugs and corticosteroids at the equal dose for 2 weeks, DMARDs such as methotrexate and sulfasalazine for 12 weeks before the start of the study and for the duration of the study, and without corticosteroids administered parenterally within 4 weeks before and during the study. The exclusion criteria were treatment with biological agents, contraindications to cryotherapy, and refusal to participate in the study. All patients were nonsmokers.

The baseline characteristics of patients are shown in Table 1.

### 2.2. Ethical Considerations

This study was approved by the Research Ethics Committee at Poznan University of Medical Sciences, under protocol number 501/15. Each participant was informed about the study and signed the informed consent statement. All procedures performed in the study were in accordance with the principles of the 1964 Declaration of Helsinki and its later amendments or comparable ethical standards.

### 2.3. Whole-Body Cryotherapy and Exercise Procedures

The study participants were randomly allocated by a physician into three groups: WBC at −110°C, WBC at −60°C, and a non-WBC group. The WBC procedure was performed in a cryogenic electric chamber which uses a compressor cooling system to produce extreme low temperature. Patients wearing in minimal clothing (a bathing suits), gloves, socks, shoes, headband covering the ears, and a surgical mask on airway were exposed to cold daily in the following format: 30 seconds in the first room (−10°C), 30 seconds in the second room (−60°C), and 3 min in the chamber (−110°C or −60°C), where they walked in circles. All groups underwent the same 30-minute exercise therapy program (individually adjusted, depending on the functional condition of the patient). It consisted of a group of exercises (general rehabilitation exercises, stretching) and free active exercises to reduce morning stiffness and mobilize the motor and circulatory systems. The series of procedures were carried out over an 8-day intervention, with a break on Saturday and Sunday. The evaluations were conducted twice, one day before the beginning and one day after the treatment, at the same time of the morning.

### 2.4. Disease Activity Parameters

We assessed disease activity using the Bath Ankylosing Spondylitis Disease Activity Index (BASDAI), which consisted of 6 questions using a numerical rating scale (0–10) to measure factors such as severity of fatigue, spinal and peripheral joint pains, localized tenderness, and morning stiffness (both qualitative and quantitative) [27]. Additionally, we evaluated the Ankylosing Spondylitis Disease Activity Score (ASDAS-CRP), which is based on patient-reported outcomes (back pain, duration of morning stiffness, patient global assessment, and peripheral joint complaints), and measured C-reactive protein (CRP) levels [27].

Fasting blood samples were taken from the antecubital vein in the morning and were centrifuged at 5000 rpm and 4°C. The blood serum was separated and stored at −80°C until the analysis.

### 2.5. Biochemical Parameters

#### 2.5.1. IL-8 and IL-17 Quantification

We used enzyme-linked immunosorbent assay (ELISA) commercially available kits (R&D Systems) to quantify IL-8 and IL-17 levels in the serum. Briefly, 50 *μ*L and 100 *μ*L of serum (for the evaluation of IL-8 and IL-17, respectively) were pipetted into well-precoated antibody. Any IL-8 and IL-17 present in the sample were bound by the immobilized antibody. After a series of washing procedures, a substrate solution was added to the wells and the color developed in proportion to the amount of IL-8 or IL-17 present in the sample. The result was expressed in pg/mL. The inter- and intra-assay coefficients of variations (CV) for IL-8 were 5.1% and 8.4%, respectively, and for IL-17 were 4.1% and 7.2%, respectively.

#### 2.5.2. Total Antioxidant Capacity Measurement

An antioxidant assay kit (Cayman Chemical) was used to measure the total antioxidant capacity of the patients' sera. The reaction is based on the ability of antioxidants in the serum sample to inhibit the oxidation of 2,2′-azino-di-(3-ethylbenzthiazoline sulphonate). Hydrogen peroxide was used to initiate the reaction. The antioxidant capacity of the sample was compared with that of Trolox, and it was calculated as Trolox equivalents. The result was expressed in mM. The inter- and intra-assay coefficients of variations (CV) were 2.8% and 3.4%, respectively.

#### 2.5.3. TBARS Measurement

For the quantitative determination of thiobarbituric acid reactive substances, the R&D commercially available assay kit was used. Briefly, 150 *μ*L of serum after acid treatment was mixed with 75 *μ*L of the thiobarbituric acid (TBA) reagent. In the presence of heat and acid, malondialdehyde (MDA) reacts with TBA to produce a colored end product that absorbs light at 530–540 nm. The result was expressed in *μ*M. The inter- and intra-assay coefficients of variations (CV) were 1.3% and 4.4%, respectively.

#### 2.5.4. CRP Concentrations

The CRP concentrations in the serum were measured using a specified analyzer Wiener lab. CB 250. The result was expressed in mg/L.

### 2.6. Statistical Analysis

The values are expressed as means, standard deviations, medians, and interquartile ranges. The Shapiro–Wilk test was used to check the data for normal distributions. The Kruskal-Wallis test was used to determine the significance of the differences between the three groups before and after therapy (Tables 1 and 2). All groups before treatment were homogenous in baseline statistics with respect to the parameters tested. To assess the significance of the differences between the first and second examinations, the Wilcoxon test was used (Table 2).

To determine the significance of the differences between the groups WBC at −110°C and non-WBC and also between WBC at −110°C and WBC at −60°C, the Mann-Whitney test was used. Moreover, correlations between variables in all patients were assessed by using the Spearman rank test. All the analyses were performed using the Statistical 10.1 package. The level of statistical significance was set at the level of 0.05.

## 3. Results

The basic characteristics of the study group are demonstrated in Table 1.

The groups did not differ significantly at baseline in terms of age, BMI, disease duration, and disease activity parameters (BASDAI and ASDAS-CRP). The disease activity scores and biochemical parameters among the three groups of patients before and after therapy are shown in Table 2.

After therapy, in groups WBC at −110°C, WBC at −60°C, and non-WBC, the patients showed significant decreases in their BASDAI scores (in all groups *P* < 0.001), ASDAS-CRP (in groups with WBC *P* < 0.001 and non-WBC *P* < 0.01), and IL-8 (in all groups *P* < 0.001). We observed a significant increase in the TBARS concentration in group WBC at −110°C (*P* < 0.001). No significant changes were observed in the levels of IL-17, TAS, and CRP in all of the investigated groups. After therapy, no significant differences were noted between the groups WBC at −110°C and non-WBC except with respect to the BASDAI (*P* = 0.041). No significant differences after therapy were noted between the WBC groups at −110°C and −60°C.

Significant, positive correlations were observed between the IL-8 and TBARS concentrations in all patients before therapy (*r* = 0.50; *P* ≤ 0.001), as well as between BASDAI and TBARS (*r* = 0.31; *P* = 0.02). Moreover, significant correlations were found between BASDAI and ASDAS (*r* = 0.64; *P* ≤ 0.001). No significant correlations were found between IL-8 and IL-17 concentrations and BASDAI, ASDAS, or CRP.

## 4. Discussion

Cryotherapy has been used in the comprehensive treatment of patients with rheumatic diseases (especially arthritis, fibromyalgia, and ankylosing spondylitis) to relieve pain and inflammatory symptoms through the regulation of the expression of cytokines [22, 28]. However, research as to the effects of low temperatures on the human body is still ongoing. It is considered that exposure to cold can stimulate the hypothalamic-pituitary-adrenal gland and sympathetic nervous system and increase the secretion of cortisol and catecholamines [29, 30]. An important role in bidirectional communication between neuroendocrine and immune system is played by cytokines [31]. It is suggested that interaction between hormones and cytokines may affect the immune response [30].

To the best of our knowledge, this is the first study that investigates the effects of whole-body cryotherapy at different temperatures, −60°C and− 110°C, on proinflammatory cytokines and oxidative stress parameters in patients with ankylosing spondylitis. In all studied groups, after therapy, we observed the decreases in BASDAI, ASDAS-CRP, and interleukin IL-8 concentration, however, not in IL-17. While comparing the therapeutic effect between treatment groups, a significantly lower BASDAI was observed after therapy in the WBC group at −110°C compared to the non-WBC group.

A reduction of disease activity after cryotherapy in rheumatic diseases has been confirmed by other authors. Recent studies have shown a reduction in the disease activity score (DAS28) in patients with RA after local and whole-body cryotherapy and the BASDAI in AS patients after WBC [23, 26, 32–34]. In our study, like that of Hirvonen et al. in the case of RA, we did not find significant differences in the BASDAI after therapy in both WBC groups: −110°C and −60°C [35].

There is a limited number of researchers who have investigated oxidative stress parameters after cryotherapy. Lipid peroxidation occurs following attacks on polyunsaturated, membrane-bound fatty acids by reactive oxygen species and thereby initiates a self-propagating chain reaction culminating in the destruction of membrane lipids and loss of cell viability [36]. In our study, an increased TBARS concentration after therapy was observed in all treated groups; however, it was found to be significant only in the WBC at −110°C group. At the same time, none of the groups showed any significant change in TAS after therapy. This interesting fact may be explained by the increased physical activity of all the study patients. All members of the treated group performed a daily set of exercises, which may have caused an increase in their metabolic rates and oxygen consumption. This condition likely induced mitochondria to produce ROS (reactive oxygen species) as a function of oxidative stress. The moderate production of free radicals may affect the antioxidant activity of the cells via the Nrf2-ARE (nuclear factor E2-related factor 2-antioxidant response element) pathway and increase the synthesis of antioxidant enzymes [37]. We suggest that the mechanism promoting the beneficial effects of whole-body cryotherapy is based on the induction of moderate oxidative stress in patients with AS during the therapy. Further studies are planned in patients with AS to investigate the activity of Nrf2-ARE-dependent antioxidant enzymes that result from hypothermic stress.

Banfi et al., summarizing the effect of WBC in athletes, suggested that the induced generation of reactive oxygen species after WBC in healthy subjects is probably caused by muscle shivering or the metabolism and intensified oxidation of catecholamines released by cold stress [24]. Research carried out by athletes showed that a single application of WBC induced oxidative stress but at a low level. Repeated treatments do not cause cumulative effects, whereas there are adaptive changes in antioxidant status when the WBC precedes or accompanies intensive training. Nonetheless, Sutkowy et al. suggested that the beneficial effects of WBC might appear in the long term, between 9 and 20 days after exposure to cold [38].

The number of publications concerning the induction of oxidative stress in unhealthy subjects after WBC is poor. In contrast to our results, Miller et al. did not report any significant changes in the level of TBARS in patients with multiple sclerosis compared to the non-WBC group among healthy subject after ten WBC sessions at −130°C [39]. Similar results were obtained by Stanek et al. [23, 26], namely, no significant changes in the malondialdehyde (MDA) concentration in plasma while comparing the group of male AS patients before and after whole-body cryotherapy at −120°C. However, as a result of WBC on the studied group, the authors showed the decrease of total oxidant status (TOS) as well as oxidative stress index (OSI) and the increase of total antioxidant capacity measured as ferric-reducing ability of plasma (FRAP) [23, 26].

The other suggestion is brought by the recent research performed on rats. Therefore, the response of the body varies according to the applied treatment temperature. Total antioxidant capacity (TAC) increased after 5 and 10 days of 1 min WBC at −60°C, and the decrease was observed at −90°C. The level of erythrocyte MDA decreased after 5 and 10 days of WBC at −60°C, and increased concentrations were measured at −90°C [40] which is in accordance with the results of this study result.

Lubkowska et al. showed that the TAS level decreased significantly after 10 sessions of WBC among healthy men [25]. Miller et al. showed that WBC treatment resulted in the increase of TAS levels among patients suffering from multiple sclerosis, but no changes among healthy controls were observed [39]. Hirvonen et al. observed that treatment using cold temperatures induced a short-term increase in total peroxyl radical-trapping antioxidant capacity (TRAP) in patients with active RA [41]. The increase was observed only during the first treatment session with whole-body cryotherapy at −110°C but not at −60°C or during local cold.

Literature concerning levels of IL-17 and IL-8 in patients with rheumatic diseases after cryotherapy or exercise therapy is very poor. IL-17 is important in the defense against extracellular bacterial and fungal infections. However, overproduction can lead to chronic inflammation and autoimmune diseases [42]. IL-17 acting synergistically with other inflammatory cytokines (TNF, IL-1b, IL-22, and IFN*γ*) can lead to increased inflammatory production mediators, such as IL-6 and IL-8 [11].

IL-8, an oxidative stress-responsive proinflammatory chemokine, is a member of the superfamily of chemokines CXC [14]. It is released from epithelial cells after particle-induced oxidative stress causing neutrophil influx and inflammation [43]. IL-8 is the primary chemoattractant to neutrophils; the transcription of which is dependent on NF-*κ*B and I*κβ* kinase (IKK) mediators that maintain cellular oxidative stress [43]. It is believed that the imbalance of the pro- and anti-inflammatory cytokines plays a role in the induction and maintenance of pain. IL-8 is one of the proinflammatory cytokines that is known to activate the sympathetic nervous system and increase nociceptive sensitivity [28].

Among all patients in the study entry, we observed significant positive correlation between IL-8 and the TBARS concentration. In our opinion, this correlation briefly describes and corresponds with the patient's condition before therapy and may suggest that disease-related factors are involved in the increased oxidative stress in AS, as suggested previously by Solmaz and Kozaci [16].

In our study, IL-8 concentration decreased significantly after therapy in all the groups. However, in opposition to the findings reported by Azevedo et al. [14], neither disease activity parameters (BASDAI and ASDAS) nor CRP correlations were observed.

At the same time, the concentration of IL-17, the other studied proinflammatory cytokine, was not changed. We have also shown no correlation between IL-17 concentration and BASDAI, ASDAS, or CRP. This is in line with other researches; no associations of serum IL-17 levels with clinical (BASDAI) and laboratory parameters (erythrocyte sedimentation rate (ESR), CRP) were found. Moreover, no significant differences between less and more active AS were found [4]. The authors explained this observation by the small sample size of the study or the heterogeneity of AS.

The idea of our study was to analyze the status of the AS patient using available biochemical markers of inflammation and oxidative stress in relation to disease activity. This global approach is necessary in the evaluation of the studied patients' status and establishing further medical treatment of the multifactor disorders like AS.

The relatively small number of studied patients is a limitation of this study; nonetheless, differences were found between the compared groups. However, our groups were identical in baseline statistics, except for sex, medications, and comorbidities. It should be noticed that inpatients, in contrary to outpatients under physiotherapy treatment, are characterized by higher disease activity and comorbidities. These factors can have an effect on the oxidative stress, antioxidant capacity, and obtained results. However, to minimize the potential, negative effect on the researched parameters, no changes in treatment were performed. The healing cycle included only 8 treatments, which resulted from the limited time of stay at the hospital ward. It cannot be ruled out that a larger number of treatments could lead to a better therapeutic effect. Long-term follow-up effects have not been studied.

An evaluation of the therapeutic effect might have brought greater understanding of the result while being analyzed from a distant perspective. However, due to the complicated etiology of the AS disorder, the need for further studies exploring the mechanism as well as the effective therapy is indisputable.

## 5. General Conclusion

WBC, especially at −110°C, had a positive effect on lowering ankylosing spondylitis disease activity (BASDAI).

The influence of WBC on changes in antioxidant status has not been demonstrated; however, further studies are needed to assess the character of TBARS changes observed in the study.

## Figures and Tables

**Table 1:** Baseline characteristics of the patients with AS.

	Group WBC −110°C with exercise therapy (*N* = 23)	Group WBC −60°C with exercise therapy (*N* = 21)	Group non-WBC with exercise therapy (*N* = 21)	*P*
Age (years)	47.7 (9.97)	50.8 (12.24)	48.4 (9.38)	0.4465
Gender M/F	21/2	17/4	13/8	
BMI	25.7 (4.5)	28.8 (4.21)	27.3 (5.59)	0.0925
Disease duration (years)	13.3 (10.88)	13.1 (12.45)	8.3 (6.87)	0.4629
Medication				
NSAID (*n*)	23	21	20	
DMARD synthetic (*n*)	11	2	5	
Glucocorticoids (*n*)	3	0	2	
Comorbidities				
Hypertension (*n*)	7	8	10	
Diabetes mellitus (*n*)	0	1	4	

The results are expressed as mean (SD). BMI: body mass index; NSAID: nonsteroidal anti-inflammatory drug; DMARD: disease-modifying antirheumatic drug.

**Table 2:** Clinical characteristics of the studied group before and after the therapy.

	Group (*N* = 23) WBC −110°C with exercise therapy			Group (*N* = 21) WBC −60°C with exercise therapy			Group (*N* = 21) non-WBC with exercise therapy			*P*_Before_ *P*_After_
	Before	After	*P*	Before	After	*P*	Before	After	*P*	
BASDAI	5.39 (1.12) (4.8; 4.6–6.5)	3.77 (1.56) (3.3; 2.6–5.1)	0.0001	5.48 (1.07) (5.5; 4.4–6.2)	3.7 (1.44) (3.2; 3.0–4.5)	0.0001	5.83 (1.29) (5.9; 4.6–6.8)	4.6 (1.72) (4.3; 3.7–5.6)	0.0007	0.4897 0.0790
ASDAS-CRP	3.17 (0.67) (3.24; 2.84–3.59)	2.66 (0.81) (2.70; 2.22–3.09)	0.0008	3.34 (0.85) (3.22; 2.69–3.94)	2.66 (0.7) (2.64; 2.26–3.03)	0.0001	3.34 (0.78) (3.38; 3.17–3.85)	2.95 (0.85) (2.88; 2.56–3.53)	0.0064	0.6467 0.4157
IL-17 (pg/m/L)	452.58 (58.76) (445.39; 397.73–486.60)	449.15 (70.30) (431.74; 397.73–486.6)	0.6378	457.83 (62.08) (445.39; 411.31–507.35)	449.34 (79.98) (424.92; 404.52–472.83)	0.3392	436.84 (50.65) (431.74; 404.52–459.09)	434.34 (58.73) (411.31; 397.73–459.09)	0.9108	0.5308 0.8118
IL-8 (pg/mL)	702.78 (705.62) (*n*∗ = 22) (413.49; 242.40–723.37)	139.1 (210.98) (29.16; 13.79–116.49)	0.0005	642.59 (625.31) (*n*∗ = 19) (500.87; 282.60–783.30)	130.26 (204.93) (37.41; 10.80–136.36)	0.0005	709.20 (724.07) (511.94; 169.47–1048.21)	42.31 (46.39) (29.19; 6.45–54.52)	0.0001	0.9917 0.3161
TAS (mM)	1.42 (1.37) (*n*∗ = 22) (1.43; 0.85–1.87)	1.50 (1.70) (1.02; 0.7–1.71)	0.5922	1.84 (1.18) (1.43; 1.15–2.55)	1.82 (1.5) (1.3; 0.82–2.29)	0.6894	1.47 (1.22) (*n*∗ = 20) (1.36; 0.73–2.24)	1.41 (0.82) (1.18; 0.87–2.11)	0.7938	0.6552 0.3860
TBARS (*μ*M)	1.14 (0.42) (1.15; 0.79–1.42)	1.78 (0.75) (1.69; 1.3–2.43)	0.0002	1.41 (0.63) [*n*∗ = 20] (1.33; 1.11–1.67)	1.61 (0.86) (1.46; 1.05–1.85)	0.0910	1.36 (0.43) (*n*∗ = 19) (1.4; 1.24–1.64)	1.54 (0.68) (1.52; 0.97–1.97)	0.3341	0.1472 0.3308
CRP (mg/L)	8.85 (13.34) (5.0; 2.8–7.5)	8.5 (11.48) (6.0; 3.52–8.66)	0.6951	10.75 (11.76) (7.02; 4.5–13.50)	9.67 (8.82) (7.5; 3.4–11.31)	0.5755	7.54 (5.64) (7.0; 4.0–10.2)	8.29 (8.29) (6.64; 4.5–8.99)	0.6009	0.3507 0.7222

The results are expressed as mean (SD) (median; interquartile range). *P* < 0.05 is regarded as a statistically significant difference. BASDAI: Bath Ankylosing Spondylitis Disease Activity Index; ASDAS: Ankylosing Spondylitis Disease Activity Score; IL: interleukin; TAS: total antioxidant status; TBARS: thiobarbituric acid reactive substances; CRP: C-reactive protein; *n*^∗^: adjusted number of subjects; WBC: whole-body cryotherapy.

## Data Availability

All data are included in the tables within the article.

## References

[1] Braun J., Sieper J. (2007). Ankylosing spondylitis. *The Lancet*.

[2] Smith J. A. (2015). Update on ankylosing spondylitis: current concepts in pathogenesis. *Current Allergy and Asthma Reports*.

[3] Zhong Y., Zhang B., Liao Z., Gu J. (2016). Assessment of ankylosing spondylitis by serum cytokine profile. *International Journal of Clinical and Experimental Medicine*.

[4] Mei Y., Pan F., Gao J. (2011). Increased serum IL-17 and IL-23 in the patient with ankylosing spondylitis. *Clinical Rheumatology*.

[5] Kozaci L. D., Sari I., Alacacioglu A., Akar S., Akkoc N. (2010). Evaluation of inflammation and oxidative stress in ankylosing spondylitis: a role for macrophage migration inhibitory factor. *Modern Rheumatology*.

[6] Straburzyńska-Lupa A., Nowak A., Romanowski W., Korman P., Pilaczyńska-Szcześniak Ł. (2013). A study of the link between bone turnover markers and bone mineral density with inflammation and body mass in postmenopausal women with active rheumatoid arthritis. *Journal of Bone and Mineral Metabolism*.

[7] Wang X., Lin Z., Wei Q., Jiang Y., Gu J. (2009). Expression of IL-23 and IL-17 and effect of IL-23 on IL-17 production in ankylosing spondylitis. *Rheumatology International*.

[8] Wendling D. (2010). IL-23 and IL-17 in ankylosing spondylitis. *Rheumatology International*.

[9] Jethwa H., Bowness P. (2016). The interleukin (IL)-23/IL-17 axis in ankylosing spondylitis: new advances and potentials for treatment. *Clinical & Experimental Immunology*.

[10] Miossec P. (2009). IL-17 and Th17 cells in human inflammatory diseases. *Microbes and Infection*.

[11] Beringer A., Noack M., Miossec P. (2016). IL-17 in chronic inflammation: from discovery to targeting. *Trends in Molecular Medicine*.

[12] Skov L., Beurskens F. J., Zachariae C. O. C. (2008). IL-8 as antibody therapeutic target in inflammatory diseases: reduction of clinical activity in palmoplantar pustulosis. *Journal of Immunology*.

[13] Limón-Camacho L., Vargas-Rojas M. I., Vázquez-Mellado J. (2012). *In vivo* peripheral blood proinflammatory T cells in patients with ankylosing spondylitis. *The Journal of Rheumatology*.

[14] Azevedo V. F., Faria-Neto J. R., Stinghen A. (2013). IL-8 but not other biomarkers of endothelial damage is associated with disease activity in patients with ankylosing spondylitis without treatment with anti-TNF agents. *Rheumatology International*.

[15] Karakoc M., Altindag O., Keles H., Soran N., Selek S. (2007). Serum oxidative–antioxidative status in patients with ankylosing spondilitis. *Rheumatology International*.

[16] Solmaz D., Kozaci D., Sari I. (2016). Oxidative stress and related factors in patients with ankylosing spondylitis. *European Journal of Rheumatology*.

[17] Stanek A., Cieślar G., Romuk E. (2010). Decrease in antioxidant status of plasma and erythrocytes from patients with ankylosing spondylitis. *Clinical Biochemistry*.

[18] Guillot X., Tordi N., Mourot L. (2014). Cryotherapy in inflammatory rheumatic diseases: a systematic review. *Expert Review of Clinical Immunology*.

[19] Bouzigon R., Grappe F., Ravier G., Dugue B. (2016). Whole- and partial-body cryostimulation/cryotherapy: current technologies and practical applications. *Journal of Thermal Biology*.

[20] Braun K. P., Brookman-Amissah S., Geissler K., Ast D., May M., Ernst H. (2009). Whole-body cryotherapy in patients with inflammatory rheumatic disease. A prospective study. *Medizinische Klinik*.

[21] Lubkowska A., Szyguła Z., Chlubek D., Banfi G. (2011). The effect of prolonged whole-body cryostimulation treatment with different amounts of sessions on chosen pro- and anti-inflammatory cytokines levels in healthy men. *Scandinavian Journal of Clinical and Laboratory Investigation*.

[22] Stanek A., Cieślar G., Strzelczyk J. (2010). Influence of cryogenic temperatures on inflammatory markers in patients with ankylosing spondylitis. *Polish Journal of Environmental Studies*.

[23] Stanek A., Cholewka A., Wielkoszyński T., Romuk E., Sieroń A. (2018). Whole-body cryotherapy decreases the levels of inflammatory, oxidative stress, and atherosclerosis plaque markers in male patients with active-phase ankylosing spondylitis in the absence of classical cardiovascular risk factors. *Mediators of Inflammation*.

[24] Banfi G., Lombardi G., Colombini A., Melegati G. (2010). Whole-body cryotherapy in athletes. *Sports Medicine*.

[25] Lubkowska A., Szygula Z., Klimek A. J., Torii M. (2010). Do sessions of cryostimulation have influence on white blood cell count, level of IL6 and total oxidative and antioxidative status in healthy men?. *European Journal of Applied Physiology*.

[26] Stanek A., Cholewka A., Wielkoszyński T., Romuk E., Sieroń A. (2018). Decreased oxidative stress in male patients with active phase ankylosing spondylitis who underwent whole-body cryotherapy in closed cryochamber. *Oxidative Medicine and Cellular Longevity*.

[27] Sieper J., Rudwaleit M., Baraliakos X. (2009). The Assessment of SpondyloArthritis international Society (ASAS) handbook: a guide to assess spondyloarthritis. *Annals of the Rheumatic Diseases*.

[28] Bettoni L., Bonomi F. G., Zani V. (2013). Effects of 15 consecutive cryotherapy sessions on the clinical output of fibromyalgic patients. *Clinical Rheumatology*.

[29] Marino F., Sockler J. M., Fry J. M. (1998). Thermoregulatory, metabolic and sympathoadrenal responses to repeated brief exposure to cold. *Scandinavian Journal of Clinical and Laboratory Investigation*.

[30] Rhind S. G., Castellani J. W., Brenner I. K. M. (2001). Intracellular monocyte and serum cytokine expression is modulated by exhausting exercise and cold exposure. *American Journal of Physiology-Regulatory, Integrative and Comparative Physiology*.

[31] Felten S. Y., Madden K. S., Bellinger D. L., Kruszewska B., Moynihan J. A., Felten D. L. (1998). The role of the sympathetic nervous system in the modulation of immune responses. *Advances in Pharmacology*.

[32] Gizińska M., Rutkowski R., Romanowski W., Lewandowski J., Straburzyńska-Lupa A. (2015). Effects of whole-body cryotherapy in comparison with other physical modalities used with kinesitherapy in rheumatoid arthritis. *BioMed Research International*.

[33] Jastrząbek R., Straburzyńska-Lupa A., Rutkowski R., Romanowski W. (2013). Effects of different local cryotherapies on systemic levels of TNF-*α*, IL-6, and clinical parameters in active rheumatoid arthritis. *Rheumatology International*.

[34] Stanek A., Cholewka A., Gadula J., Drzazga Z., Sieron A., Sieron-Stoltny K. (2015). Can whole-body cryotherapy with subsequent kinesiotherapy procedures in closed type cryogenic chamber improve BASDAI, BASFI, and some spine mobility parameters and decrease pain intensity in patients with ankylosing spondylitis?. *BioMed Research International*.

[35] Hirvonen H. E., Mikkelsson M. K., Kautiainen H., Pohjolainen T.H., Leirisalo-Repo M. (2006). Effectiveness of different cryotherapies on pain and disease activity in active rheumatoid arthritis. A randomised single blinded controlled trial. *Clinical and Experimental Rheumatology*.

[36] Datta S., Kundu S., Ghosh P., De S., Ghosh A., Chatterjee M. (2014). Correlation of oxidant status with oxidative tissue damage in patients with rheumatoid arthritis. *Clinical Rheumatology*.

[37] Nguyen T., Nioi P., Pickett C. B. (2009). The Nrf2-antioxidant response element signaling pathway and its activation by oxidative stress. *The Journal of Biological Chemistry*.

[38] Sutkowy P., Augustyńska B., Woźniak A., Rakowski A. (2014). Physical exercise combined with whole-body cryotherapy in evaluating the level of lipid peroxidation products and other oxidant stress indicators in kayakers. *Oxidative Medicine and Cellular Longevity*.

[39] Miller E., Mrowicka M., Malinowska K., Zolynski K., Kedziora J. (2010). Effects of the whole-body cryotherapy on a total antioxidative status and activities of some antioxidative enzymes in blood of patients with multiple sclerosis-preliminary study. *The Journal of Medical Investigation*.

[40] Skrzep-Poloczek B., Romuk E., Wiśnowiska B. (2017). Effect of whole-body cryotherapy on antioxidant systems in experimental rat model. *Oxidative Medicine and Cellular Longevity*.

[41] Hirvonen H., Kautiainen H., Moilanen E., Mikkelsson M., Leirisalo-Repo M. (2017). The effect of cryotherapy on total antioxidative capacity in patients with active seropositive rheumatoid arthritis. *Rheumatology International*.

[42] Konya C., Paz Z., Apostolidis S. A., Tsokos G. C. (2015). Update on the role of interleukin 17 in rheumatologic autoimmune diseases. *Cytokine*.

[43] Qazi B. S., Tang K., Qazi A. (2011). Recent advances in underlying pathologies provide insight into interleukin-8 expression-mediated inflammation and angiogenesis. *International Journal of Inflammation*.

